# PHYSICAL ASSESSMENT IN SURFERS: GUIDELINES FOR HEALTH PROFESSIONALS - PART 1 UPPER QUARTER

**DOI:** 10.1590/1413-785220253304e290571

**Published:** 2025-09-08

**Authors:** Guilherme Vieira Lima, Eduardo Takeuchi, Pedro Miguel Carriço de Seixas, Alexander Rehder, Marcus Vinicius Pereira Prada, Marcelo Baboghluian, Guilherme Carlos Brech

**Affiliations:** 1 Grupo de Estudos do SID (Surf Information Data), Sao Paulo, SP, Brazil.; 2 Personal Boards, Sao Paulo, SP, Brazil.; 3 Faculdade de Medicina do ABC, Departamento de Ortopedia e Traumatologia, Cirurgia do Ombro e Cotovelo, Santo Andre, Sao Paulo, SP, Brazil.; 4 Surfing Medicine International (SMI), Netherlands.; 5 Escola Superior de Saude Atlantica, Oeiras, Portugal.; 6 Universidade de Sao Paulo (USP), Faculdade de Medicina, Departamento de Ortopedia e Traumatologia, Laboratorio de Estudos do Movimento, Sao Paulo, SP, Brazil.; 7 Universidade Sao Judas Tadeu, Programa de Pos-Graduacao em Ciencias do Envelhecimento, Sao Paulo, SP, Brazil.

**Keywords:** Practice Guideline, Water Sports, Musculoskeletal Pain, Shoulder, Spine, Guia de Prática Clínica, Esportes Aquáticos, Dor Musculoesquelética, Ombro, Coluna Vertebral

## Abstract

In Brazil, surfing has gained popularity in recent decades, driven by beautiful beaches, good wave conditions and the spirit of adventure that permeates Brazilian culture, but also increasing the risk of injuries. During surfing, the surfer spends most of the time lying prone on the board while paddling, placing heavy demands on the musculoskeletal system in the cervical and lumbar spine, as well as in the shoulder region, which are important points for complaints of chronic injuries among surfers. The objective of the present study was to update musculoskeletal assessment of the upper quarter, related to physical examination and functional tests that can be applied to surfers. This is an update study based on an integrative review through a bibliographical survey in which national and international journals indexed in the scientific databases Scielo and PubMed were evaluated, developed and analyzed by a group of experts in the area of surf medicine and health composed of physical educator, physiotherapists and sports doctors. This guideline study compiled important information regarding the prevalence of upper quarter musculoskeletal injuries in surfers, guiding the surfer's outpatient assessment, considering the specificity of the sport and biomechanical gesture involved. ***Level of Evidence III; Expert opinion.***

## RESUMO

No Brasil, o surfe tem ganhado popularidade nas últimas décadas, impulsionado pelas belas praias, boas condições de ondas e o espírito de aventura que permeia a cultura brasileira, aumentando também o risco de lesões. Durante a prática de surfe, o surfista permanece grande parte do tempo, deitado sobre a prancha remando, exigindo uma demanda acentuada do sistema musculoesquelético na região da coluna cervical e lombar, assim como na região dos ombros, pontos importantes de manifestação de queixas de lesões crônicas dos surfistas. O objetivo do presente estudo foi realizar uma atualização referente aos estudos relacionados à avaliação musculoesquelética do quadrante superior, relacionada ao exame físico e testes funcionais que possam ser aplicados ao surfista. Trata-se de estudo de atualização baseado em uma revisão integrativa através de levantamento bibliográfico no qual foram avaliados periódicos nacionais e internacionais indexados nas bases científicas Scielo e PubMed, desenvolvida e analisada por um grupo de especialistas na área de medicina e saúde do surfe composta por educador físico, fisioterapeutas e médicos do esporte. Este estudo de diretrizes compilou informações importantes referente a prevalência de lesões musculoesqueléticas do quadrante superior no surfista, norteando a avaliação ambulatorial do surfista, pensando na especificidade da modalidade e gesto biomecânico envolvido. ***Nível de Evidência III; Opinião do especialista.***

## INTRODUCTION

Surfing is a sport practiced by different age groups, genders with different levels of performance (from beginners to professionals), locations and environmental conditions. This sport is in full growth, and although it is difficult to define the exact number of practitioners in the world^1^ it is estimated that there are approximately 30 to 37 million surfers around the world,^2,3^ with a significant increase in the number of surfers in the world and especially in Brazil, which also increases the risk of injuries.^4^ In Brazil, surfing has gained significant popularity in recent decades, driven by the beautiful beaches, good wave conditions and the spirit of adventure that permeates the Brazilian culture. The popularity of surfing has grown rapidly in recent years, boosted by the sport's debut at the Tokyo Olympic Games in 2020.^5^

In the competitive "world of surfing", Brazil is a country that has been standing out, with the "Brazilian Storm" (the group of Brazilian surfers competing the world circuit) achieving expressive results in the last 10 years. In facto, the Brazilians have won 6 world titles and were the first country to win an Olympic gold in Surfing. This fact has also contributed to the growth of sport in Brazil.^6^ In addition, the country today has three wave pools in full operation, with two others under construction, and it is estimated that it will soon be the country with the largest number of wave pools in the world.^7^

During the practice of surfing, the surfer spends most of the time (more than 60%), lying prone on the surfboard. This position, similar to the crawl swimming position, but relies on using a rigid surface in the ventral region (the board), which requires an increased angle of extension of the cervical and lumbar spine and movements of the upper limbs also with specifics of the sport.^8^ During this time of practice there is a strong demand for the musculoskeletal system in the cervical and lumbar spine, as well as in the shoulder region, which are important points of manifestation of complaints related to surfers’ chronic injuries.^5,9^

Thus, it becomes important to set guidelines that can help in the guidance of evidence-based clinical practice, for the musculoskeletal evaluation in the surfer's outpatient environment. These should involve physical examination and functional tests of the upper quarter, such training load control, evaluation of treatment and performance, as well as preventive measures to minimize injury risks.

In this sense, the aim of this study was to carry out an update regarding the studies related to musculoskeletal evaluation of the upper quarter, concerning physical examination and functional tests that can be applied to the surfer.

## METHODS

This is an update study in which a bibliographic survey was conducted between June 2023 and July 2024 on Scielo and PubMed platforms, with terms related to surfing. However, as the scientific production on the topic is still quite scarce, the search has been widened to support the elaboration of a physical evaluation and functional tests guideline specific for surfers’ upper quarter assessement.

### Surfer's musculoskeletal evaluation

#### Clinical Surfer Assessment

The clinical evaluation before participation in sports is the subject of long debates given the controversy of its effectiveness in detecting conditions that predispose people to diseases or injuries.^10^ However, important entities recommend, and even require, the evaluation before participation.^11^

In this sense, the periodic clinical evaluation is an indispensable tool for improving performance, disease and injury prevention. When related to surfers, it becomes even more relevant since the characteristics of the practice often expose the individual to extreme weather conditions (e.g. heat, cold, wind), saltwater and long periods of dehydration.^12^

Thus, the objective of periodic clinical evaluations, at any age, should be to determine the physiological and psychological health of the practitioner, seeking to identify risk factors and opportunities for health improvement,^13,14^ serving as a basis for guidance in the areas of training, nutrition and psychology.

Biomarkers are gaining evidence and being used for both professional athletes and recreational practitioners, in order to identify injury risks and improvements in training and recovery.^15,16^

For professional athletes, lung function test are important for determining maximum respiratory pressures directly linked to CO2 tolerance and fatigue.^17^ In addition, clinical investigation of cardiomyopathies, valvulopathies, hypertension, diabetes, hormonal imbalances, anemias, are some of the clinical conditions that should be evaluated at all ages in identifying risk factors for injuries as well as performance improvement.^14^

#### Musculoskeletal evaluation of the surfer in an outpatient environment

##### Spine

- **Biomechanics**
  Postural dysfunctions of surfers in the spine are mainly associated with the biomechanical gesture of paddling, but also have a relationship with the rotational movements of the trunk involved in the manoeuvres. Paddling is responsible for about 55% of the time spent during a surfing session^18^ and the prone position on the board requires a high demand from the spine.
  Although surfing is a sport that demands a lot of postural control, since staying on the board in the water generates a lot of instability, there is no consensus regarding the fact that the level of the surfer presents greater postural control. Chapman's and colleagues study,^19^ found that the values of postural oscillation do not provide clear evidence as to whether surfing experience facilitates adjustments in the postural control system. Even when comparing surfers and non-surfers, no evidence of changes in postural balance is found.^20^ However, simultaneous findings of mental tasks illustrate that there may be systematic differences in balance skills between experienced surfers and the control group. In the study of Paillard and colleagues,^21^ it was found that professional surfers present greater sensory motor domain using vision to maintain postural control when compared with amateur surfers, despite being a study with a small sample.
- **Epidemiology and evaluation of vertebral lesions**
  Spine injuries are common and account for 16 to 20% of all surfer chronic injuries.^22,23^
- **Structures at Risk**
  Structures involved in the risk of dysfunction of the spine are mainly associated with the surfer's paddling gesture, which involves an extension with rotation of the spine during paddling, with the lumbar and cervical segments being the most affected.^24^
  However, Furness and colleagues^25^ highlight that the thoracic spine is a key area that is overloaded, especially considering when the amplitude of movement is reduced and can result in stress in surrounding joints and potentially affect surfing performance.^25^
  As for the structures that can be affected in these segments, we can highlight from muscle groups responsible for the extension of the trunk and the height of the head,^26^ as well as structures related to the joint complex, disc-vertebral joints to the facetaries. These may or may not be associated with some root manifestation due to compression of nerve tissue.^24^
- **Assessment**
  The postural evaluation itself is a very subjective and qualitative evaluation. It should be guided towards the evaluation of deformities, mobility tests, evaluation of overload of posterior structures/ listesis and clinically most relevant neurological tests. In this sense, the evaluation should involve other segments such as hip and shoulder, mainly due to the relationship with the disfunctions of the lumbar spine and cervical, respectively.
  If the surfer shows a complaint of acute lumbar pain and/or root component, the clinical history should be collected and the clinical examination based on alarm signs ("flags") should be adopted to guide the evaluation, and consequently the treatment.^27^
  The evaluation of the mobility of the spine and shoulders is extremely important in the surfer due to the paddling gesture that occurs during most of the time of practice. The flexibility of the lumbar spine can be measured using the modified Schober test (distance between the lumbosacral junction and 10 cm above), and a variation equal to or greater than 5 cm is considered normal. Intra-observer intra-class correlation coefficient (ICC) is 0.96 inter-observer 0.93, indicating high reliability.^28^ Furness and colleagues^29^ highlight the importance of assessing the mobility of the thoracic spine in the sagital plane, revealing excellent intra-evaluator reliability values for lumbar blockage with the thoracic rotation method. Poor mobility of the thoracic spine can be associated with increased cervical and lumbar pain^30^ and shoulder dysfunction.^31^
  The physical examination of the surfer's spine should take into account the investigation of possible overload and/or listesis dysfunctions. In the physical examination of these patients, lumbar hyperlordosis and shortening of the posterior musculature of the thigh might be present; in addition, pain complaints in the spine, radiating to the lower limbs may also be found, which are frequent symptoms of this dysfunction, and can also be aggravated with the extension of the lumbar spine or with extension of the lower limbs. In the palpation of the thinning processes it is possible to identify some gap, when there is the presence of some slip.^32^
  In addition, neurological tests become important when a) there's a suspecting discopathy, both of the cervical and lumbar spine, which may manifest through some root symptom if positive; or b) in a preventive way to evaluate any possible manifestation of some symptom of this nature, even if in the initial stages.^33,34^
- **Other injury to the spine**
  Surfers’ myelopathy (SM) is a rare but associated vertebral dysfunction.^33,34^ SM can be considered a severe acute injury, characterized by a rare, non-traumatic spinal cord injury associated with hyper-extension of the spine. It is estimated that the incidence of MS ranges from 2.2 to 6.6 injuries per 1,000 people who surf. Myelopathy is generally defined as any neurological deficit related to spinal cord dysfunction.^33^ Although little is known about this dysfunction, it is believed to occur in beginners, healthy surfers, without previous spinal or vascular problems. There is a decrease in strength and sensitivity of the lower limbs, which can affect bladder/intestinal function and walking. In most cases, this neurological deficit is transient, but it is serious and requires urgent care.^33,34^
  In this sense, in addition to the lumbar and cervical hyperextension related to the surfer's posture, due to the gesture of paddling, other postural changes can be present. For example, the anterior deviation of the shoulders due to pronounced internal rotation, which may also be associated to the pectoralis minor shortening. Due to the need for hyper-extension to elevate the trunk and paddle on the board, the lumbar spine can often present an increase in the physiological curve (lordosis). Therefore, because of paddling, lumbar and cervical spine are areas of complaints for surferes.^4^
  Studies point to the importance of specific exercises for the CORE muscles (trunk and spine stabilizers) in the surfer for stabilization of the lumbar spine and trunk,^35^ as a strategy for the prevention of lumbalgia and cervicalgia, due to injuries associated with remade position (chronic) and maneuvers (acute) during the practice of sport.

##### Shoulder

- **Biomechanics**
  The main mechanism of injury is paddling, due to overuse, but there are many reports of acute injuries resulting in a dislocation of the glenohumeral, and in these cases, the responsible mechanism is the fall with the upper limb in abduction and external rotation with indirect trauma of the joint.^3,9,36,37^
  Paddling varies greatly in terms of the frequency and intensity of the movements. However, when considering overuse injuries related to this movement, swimming is often used as a reference for analysis and treatment. Nevertheless, we must be aware of some important differences in these two sports. The first is the unpredictability of the number, frequency and intensity of paddling in surfing; another is the position adopted by the surfer, who's on a board, in a position that promotes greater trunk extension – particulary in the thoracic and cervical regions. This position results in a shorter stroke with an entry into the water early, less glide, and consequently reduced propulsion. Moreover, it limits the athlete's ability to utilize the trunk rotation (hip roll), a movement commonly employed by swimmers to facilitate a greater stroke amplitude.^8,26^
  The causes of the biomechanical injuries are mainly: muscle imbalance, scapulo-thoracic dyscinesia, subacromial impact and internal impact.^8^
  Testing cervical and thoracic mobility is an important ally in the evaluation of the surfer, being simple tests of movement amplitude or postural correction that have as response to the improvement in some mentioned sign or symptom. A clear example that can contribute to shoulder discomfort is the decrease in the amplitude of thoracic extension, resulting in a reduction or alteration of the expected escapular movement, increasing the risk of impact around the shoulder joint.^8,38^
  Nessler and colleagues^39^ conducted a study aiming to evaluate the effects of using neoprene clothing on paddling during surfing. After the analysis of kinematics data, they concluded that the use of neoprene clothing can improve the athlete's finish technique, in addition to providing improved proprioception.
- **Epidemiology**
  Shoulder injuries are among the most common in surfers, most often of chronic origin, with an incidence of 22.4% for both professional and recreational surfers.^25^ In a study that investigated the prevalence of self-reported injuries in Australian surfers (n=685), the main shoulder injuries are highlighted: unspecific causes (6.2%); dislocation (3.6%); rotator cuff injury (1.5%); laceration (0.8%); acromioclavicular injury (0.5%) and fractures (0.5%).^40^
- **Structures at Risk**
  The main structures involved surf paddling are the muscles of the rotator cuff, pectoralis major, deltoid, latissimus dorsal, triceps and trapezius.^8^
  The functional evaluation aims to evaluate the function or performance of a body segment, and thinking about an evaluation in the surf we have to know which muscle groups are most sought after and some combinations of movement during the remade that are most recruited in this sporting gesture, which can lead to possible overloads and dysfunctions.
  The muscles that are active during remade are mainly internal rotators and shoulder flexors. Surfers with complaints in this region appear to present a decrease in the amplitude of movement and lateral shoulder rotation strength. The muscles that are involved in the internal rotation of the shoulder can be shortened, leading to aberrant scapular tilt and lateral rotation. Further research should address the potential shortening of the pectoralis minor in this dysfunction, resulting in scapular dyskinesia and subacromial pain in surfers.^8^
  Testing the most active muscles during the propulsion of the remade is a good evaluation strategy, being the muscles: chest, large dorsal, brachial triceps and deltoid, specifically the deltoid exercises greater activation at the beginning of the propulsion movement.^26^
- **Assessment**
  During the evaluation of the shoulder complex, it is important to address beyond the joints that are part of it, including the glenohumeral, the acromioclavicular and the sternoclavicular joints. In addition, it is also relevant to evaluate the scapulo-thoracic joint, the subacromial joint and adjacent joints such as the thoracic and cervical.^41^

### Special tests

Some tests may be provocative for subacromial pain, and among them are traditional tests for subacromial impact: Jobe (empty blade); Hawkins; Neer 1 and 2; Arc Painful and Lateral Rotation with resistance. Better accuracy if 3 of these 5 tests are positive.^42^

The following combinations and test results should be considered and analyzed: Empty blade (pain) - Full blade (no pain) = cinematic conflict without involvement of the Rotor Blade;^43,44^ Empty blade and full blade + (pain) Rotor blade involvement in the painful shoulder.^43,44^

For shoulder instability, the following tests should be considered: provocative - Apprehension test - Relocation test; loose - Load & Shift (previous drawer) - Sulcus sign. However, to evaluate disorders related to the brachial biceps (SLAP lesion) are indicated the tests: Speeds test; Teste O’ Brien; Biceps-Load Test.^45^

A joint assessment of strength, balance and neuromuscular control is required through clinical and functional tests: Glenoumeral Internal Rotation Test (GIRD); Lesser Breast Flexibility Test; Manual Dynamometry Tests for external (lateral) and internal (medial) shoulder rotators: due to weakness findings of lateral shoulder rotators in surfers, the evaluation of this movement becomes an interesting strategy, and manual dynamometry apparatus is required.^8^

The position of the surfer that best correlates the gesture of paddling is in ventral decubitus, since it is described in the literature there is no significant difference between the positions of measurement of lateral rotation.^46^

The scapulo-humeral rhythm (SHR) should consider the movement of the scapula during shoulder flexion/abduction movements with load, considering the body weight parameters of the patient with the added load on the upper limb to be tested, being people up to 68 kg = 2kg; people >68 kg = 3kg.^47^ With shoulder flexion, the changes in the SHR are more obvious. Any obvious escapular dyskinesia such as winged scapula, excessive elevation and anterior *tilt*, may be related to shoulder dysfunction. This test should always be related to other tests and patients´ characteristics. It is important to assess and classify both scapulas as: normal, subtle dyskinesia, obvious dyskinesia.^47^

#### Elbow

- **Epidemiology and Biomechanics**
  Surfing elbow and forearm injuries are uncommon and account for an incidence of 3 to 6% of all injuries. There are no specific studies for the mechanism of injury of this segment, but they are probably related to the act of paddling, riding the wave and getting up on the board.^37,48^
- **Structures at risk**
  The type of injury is mostly tendinopathy and the structures most commonly involved are the tendons of the muscles: triceps, finger extensors and wrist, biceps brachial.^37^
- **Assessment**
  According to the main complaints, it becomes important to perform palpation of the following structures: medial epicondylum; lateral epicondylum; olecrane and tendon of the triceps; and biceps tendon. In case of pain during palpation (in non-traumatic cases), probably some inflammatory process is involved. Note: in case of painful palpation associated with symptoms such as shock, tingling, it is important to investigate compressive nerve syndromes. The Tinel test is valid for these situations. In addition, other special tests can be indicated in the evaluation of the elbow such as that of Mill and Cozen and the Golfer's Elbow, and it is important to also evaluate the muscle strength of pronation/ supination/ flexion and elbow extension/ flexion and extension of the handle, for this one can use a manual dynamometer.^49^

### Hand and wrist

Hand and wrist injuries are uncommon during surfing. They range from 2.4 to 4.1% of all acute injuries and are related to direct trauma against the board itself or seabed floor.^23^

The most common types of injuries are contusions, fractures, dislocations, and small wounds. The work of Rujis and colleagues ^50^ showed the pattern of injuries occurring in the fingers of the hands due to compression caused by the "leash" when wrapped and strained in the finger, while the board is being pulled by the wave force. Our literature research found no reports regarding surfing practice and chronic hand and wrist injuries.

### Functional tests

#### Mobility tests

The amplitude of the shoulder's medial rotation movement is important to be evaluated as a medial rotation deficit <20% may increase the risks of injury for athletes who practice overhead sports.^51^ This test has an intra-examinator correlation coefficient (ICC) of 0.85.^52^

The Leg Lateral Reach Test (LLRT) is a test used to evaluate the mobility of the toracolombar region. The test is performed with the surfer in dorsal decubitus, keeping the arms on the ground next to the trunk and raise the leg to be tested to the contralateral side seeking the greatest possible reach, without pulling the scapulas from the ground. The test has an ICC of 0.97 inter-examiners and 0.99 intra-examiners.^53^

#### Muscle Strength and Stability Tests

**Figure 1:**
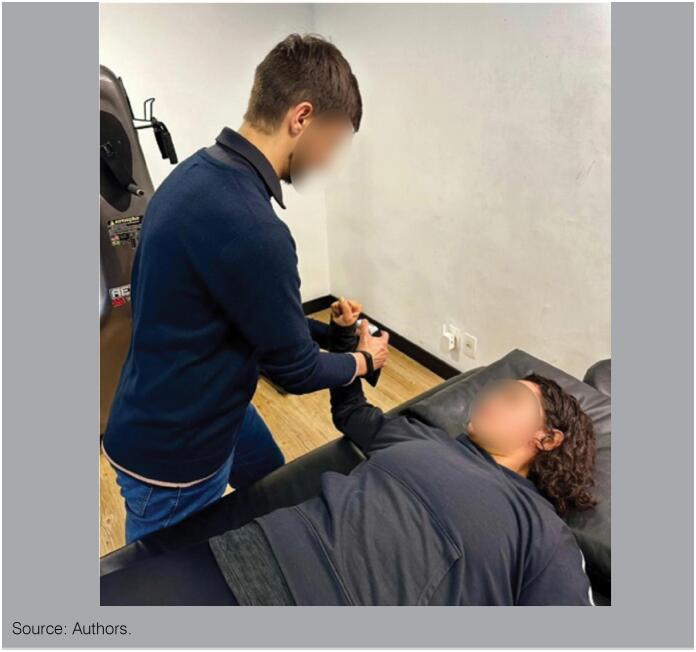
Positioning for the evaluation of the isometric strength of the side rotators with dynamometer.

The evaluation of the isometric muscle strength of the lateral shoulder rotators is also important due to the surfer's paddling gesture. The test has an ICC of 0.88 for the non-dominant side and an ICC of 0.86 for the dominant side.^44^ It is recommended to use an isometric dynamometer, evaluating with the patient in dorsal decubitus. Maximum strength should be evaluated for 5 seconds with a 30-second rest interval. 3 collections should be performed and made the average for final result.^52^ (Figure 1)

**Figure 2:**
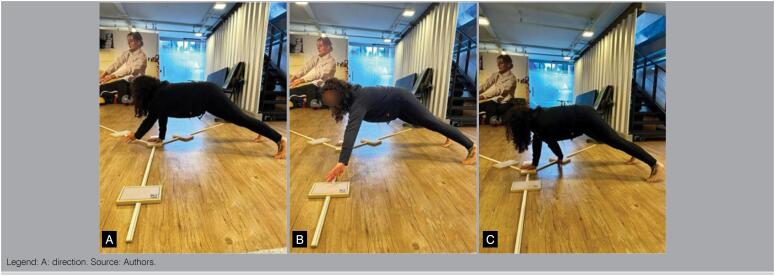
Modified Upper Quarter Y Balance Test.

The Modified Upper Quarter Y-Balance Test (mUQYBT) is a functional test that evaluates the stability and mobility of the upper limbs, unilaterally, in order to identify asymmetries that may place individuals at risk of a injury.^54^ The range of the upper limbs in three directions is evaluated: medial, laterosuperior and laterosinferior. If it is necessary to normalize the length of the limb to perform the test calculation.^44^ (Figure 2)

**Figure 3:**
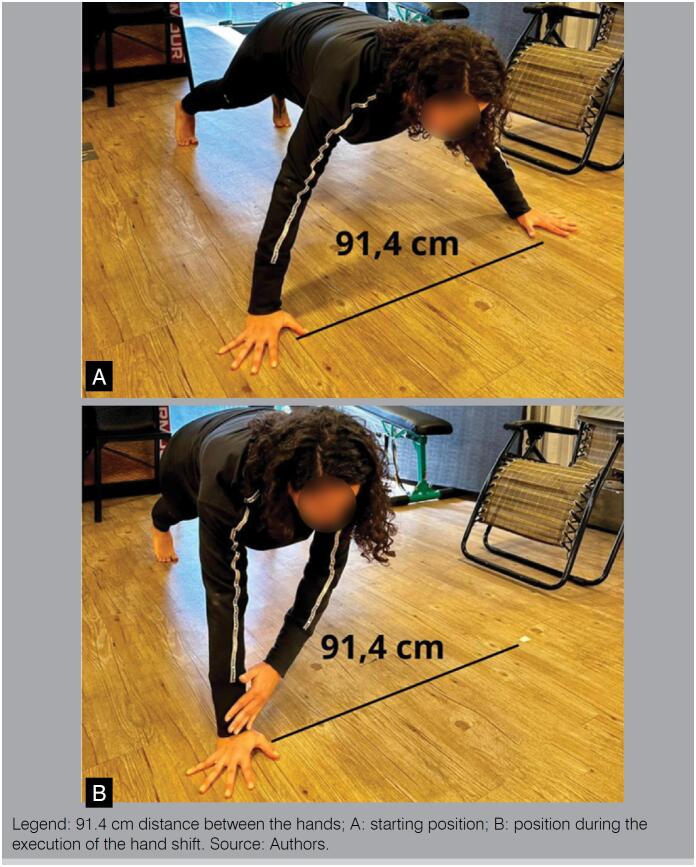
Closed Kinetic Chain Upper Extremity Stability test (CKCUEST).

The Closed Kinetic Chain Upper Extremity Stability Test (CKCUEST) is an evaluation test for dynamic stability and muscle power of the upper limbs in closed kinetic chain, and can be used for both comparative purposes (used for treatment evolution), as well as potentially predictors of injury risk, being scores classified as bad (<21 repetitions), and good (>21 repetitions).^55^ An intra-examiner intraclass correlation coefficient (ICC) of 0.92 was observed. (Figure 3)

According to Barbosa and colleagues,^56^ systematic review, one can identify that the CKCUEST presented sufficient intersession and intrasession reliability, based on evidence of moderate quality.

#### Surfist Performance Level

When we talk about surfers, the same can be considered from a child who practices the sport in a recreational way, or even amateur (Grommets), where we need to be alert to avoid an early specialization in the modality, always seeking to the maximum development of different motor skills/repertoires,^57^ as well as adults in different age groups and levels of sporting ability. We may be dealing, in the outpatient assessment, with adult surfers or even elderly amateur (recreational), who are looking for a practice of physical activity and lifestyle, where they aim for longevity in the sport.^24^ But we can also refer to the surfer, professional athlete, who is looking for better performance and in minimizing the risk of injuries.^36^ In this sense, the direction of the musculoskeletal assessment of the surfer is directly related to the specificity of the modality, but also to the purpose of the surfer within the sport.

#### Personal and Behavioral Habits

Personal and behavioral habits in surfers at different skill levels may be related to prevention or the increased risk of musculoskeletal injury. It is well known that moderate to high intensity physical activity generates specific adaptations, and good eating habits are necessary to generate the necessary contribution for these adaptations.^58^ In addition, the hydration routine is a crucial point in surfers, since it is practiced in different environmental conditions, different clothing and different degrees of intensity, and without any water replacement during the practice.^12^ Adequate sleep promotes restoration in the immune, endocrine and nervous system, with direct action in reducing injury and performance.^59^ The routine and periodization of training is another factor to be considered since, depending on the environmental conditions, the surfers will be exposed to the technical and tactical training of the modality in greater or smaller amount.^60^

The present study presents limitations because it is a review study based on the existing but scarce on the specific topic related to surfing. However, this group of researchers and clinicians directly involved in the modality, sought to develop a guideline of clinical practices that could assist in the decision-making process for health professionals working with recreational and surfing athletes, that shows a strong growth in Brazil and the world. We highlighted, and stimulated through this study, the need for more prospective studies with better methodological quality focused on the topic of surfers physical assessment.

## FINAL CONSIDERATIONS

This guideline compiled important information concerning the prevalence of musculoskeletal injuries of the upper quarter in the surfer, guiding the surfer's outpatient evaluation, considering the specificity of the sport and biomechanical gesture involved. In addition, the need for scientific studies aimed at surf medicine becomes even more obvious.
